# Common Delayed Senescence of Melanocytes from Multiple Primary Melanoma Patients

**DOI:** 10.1016/j.jid.2016.10.026

**Published:** 2017-03

**Authors:** Jaskaren S. Kohli, Elena Tolomio, Simona Frigerio, Andrea Maurichi, Monica Rodolfo, Dorothy C. Bennett

**Affiliations:** 1 Cell Biology & Genetics Research Centre, Molecular & Clinical Sciences Research Institute, St. George’s, University of London, Cranmer Terrace, London, UK; 2 Department of Surgery, Melanoma and Sarcoma Unit, Fondazione IRCCS Istituto Nazionale Tumori, Milan, Italy; 3 Department of Experimental Oncology and Molecular Medicine, Immunotherapy Unit, Fondazione IRCCS Istituto Nazionale Tumori, Milan, Italy

**Keywords:** MPM, multiple primary melanoma, SPM, single primary melanoma

To the Editor

Approximately 5% of patients with cutaneous melanoma acquire at least one additional independent melanoma, a phenomenon known as multiple primary melanoma (MPM). Genetic factors are implicated because a family history is one of the strongest risk factors for MPM (Ferrone et al., 2005).

One study identified germline mutations in *CDKN2A* in approximately 15% of MPM patients (Monzon et al., 1998), and another reported these mutations to be four times more prevalent in MPM than in patients with single primary melanoma (SPM) (Pastorino et al., 2008). *CDKN2A*, the most common known familial melanoma gene, encodes p16, a broad-spectrum tumor suppressor and mediator of cell senescence (Aoude et al., 2015b, Bennett, 2016). Senescence is a permanent cellular arrest after extensive proliferation and telomere shortening/dysfunction, or other genotoxic stresses. p16 induces senescence by inhibiting CDK4-mediated phosphorylation of retinoblastoma-family proteins, resulting in retinoblastoma proteins binding and repressing E2F transcription factor activity, which is needed for S-phase entry in the cell cycle (Bennett, 2016). Human cells may senesce through either the p53 pathway, the p16 pathway, or both. p53 arrests cells by up-regulating another CDK inhibitor, p21 (CDKN1A). However, human melanocytes seem to senesce predominantly through p16. Oncogene (usually *BRAF*) activation in an epidermal melanocyte leads to an initial proliferation followed by senescence, generating a mole or nevus (Bennett, 2016).

Cell lifespan in vitro is the number of population doublings completed by a cell strain before replicative senescence. p16-null melanocytes display an increased lifespan but still senesce, through a mechanism involving p21 (Sviderskaya et al., 2003). Similarly, *CDKN2A* mutation carriers tend to have more large nevi than normal (representing more divisions before senescence) (Bennett, 2016), as do individuals with longer telomeres (Bataille et al., 2007). Moreover, mutations in other senescence-related genes have been identified in familial melanoma: *TERT*, *CDK4,* and genes encoding components of the telomeric cap shelterin, for example *POT1,* and *TERF2IP* (Aoude et al., 2015a, Robles-Espinoza et al., 2014, Shi et al., 2014). These observations suggested that MPM may commonly be associated with genetically defective or delayed melanocyte senescence.

This hypothesis has been tested by explanting melanocyte cultures from biopsy samples of sun-protected normal skin from MPM or SPM patients who are wild type for known melanoma-associated mutations other than in *MC1R* (see Supplementary Table S1 online). Biopsy samples were taken with written informed consent and institutional ethical approval. The use of SPM patients as control subjects excluded any confounding effect of simply developing melanoma. Melanocytes were serially passaged until they reached replicative arrest. Cumulative growth curves for all cultures are shown (Figure 1a, and see individual donor information in Supplementary Figure S1 and Supplementary Table S2 online). Melanocyte culture lifespans for each patient are included in Supplementary Table S1. Senescence was confirmed by β-galactosidase immunocytochemistry in all lines (Figure 1b). The final number of population doublings (lifespan) per culture was compared between the groups.

The mean lifespan for MPM patients’ melanocytes (16.3 doublings, n = 10) was over 4-fold higher than the mean lifespan for SPM patients’ melanocytes (3.7 doublings, n = 8), confirming our hypothesis (*P* = 0.0057). Normal adult melanocytes, grown in similar conditions, are reported to have lifespans ranging up to a maximum of 10 population doublings (Graeven and Herlyn, 1992). The maximum lifespan we observed among melanocyte cultures from SPM patients was nine doublings, suggesting little difference from healthy adult donors.

Substantial heterogeneity was observed among lifespans, especially in the MPM group. We examined the role of donor age, because telomere length shortens with age in some cell types. Conclusions have varied on the relationship between donor age and lifespans of skin fibroblasts (Cristofolo et al., 1998, Schneider and Mitsui, 1976); this has not been studied in melanocytes to our knowledge. MPM cultures showed a significant negative correlation of lifespan with donor age (*P* = 0.0003), whereas SPM cultures showed no correlation (*P* = 0.59) (Figure 2). Slopes of the two regression lines differed, highly significantly (*P* = 0.0046), concluding that the significant increase in melanocyte lifespan from MPM patients occurs independently of donor age.

Although there was no significant difference in the mean donor age between MPM and SPM patients (*P* = 0.66), there were two MPM patients younger, and one older, than all SPM patients. To ensure that the difference in correlation was not due to this greater range, these three patients were excluded from a separate analysis (see Supplementary Figure S2 online). Slopes were still significantly different (*P* = 0.028), and a significant negative correlation of lifespan with donor age was still seen only in melanocytes from MPM patients (*P* = 0.027). The mean melanocyte lifespan from MPM patients was still significantly higher than from SPM patients (*P* = 0.0064).

The lack of decrease of SPM melanocyte lifespan with donor age agrees with reports that adult epidermal melanocytes rarely divide, implying little telomere shortening. Regarding the MPM cultures, the skin biopsy samples from MPM patients were, by definition, taken after diagnosis of a second (or more) melanoma. Thus, in this group, young donors were patients who were young when their second melanoma arose, and these individuals tended to have melanocytes with longer lifespans. This again suggests genetic factors that increase both MPM susceptibility and melanocyte lifespan.

These data clearly support a frequent association of MPM with a genetic tendency for extended melanocyte lifespan. Telomere length is associated with melanoma risk (Burke et al., 2013) and nevus size (Bataille et al., 2007). Likewise, the specific melanoma-associated mutations in shelterin genes are predicted to increase telomere length (Aoude et al., 2015a, Robles-Espinoza et al., 2014, Shi et al., 2014). Individuals with longer telomeres would be expected to have melanocytes with greater replicative potential, resulting in greater culture lifespans, and larger nevi after an oncogenic mutation, as seen with *CDKN2A* defects. This would yield more cells per nevus, increasing the risk of further mutations and progression to melanoma. MPM patients would make good candidates for elucidating additional germline melanoma-susceptibility genes.

## ORCIDs

Monica Rodolfo: http://orcid.org/0000-0002-9196-0298

Dorothy C Bennett: http://orcid.org/0000-0002-3639-7527

## Conflict of Interest

The authors state no conflict of interest.

## Figures and Tables

**Figure 1:**
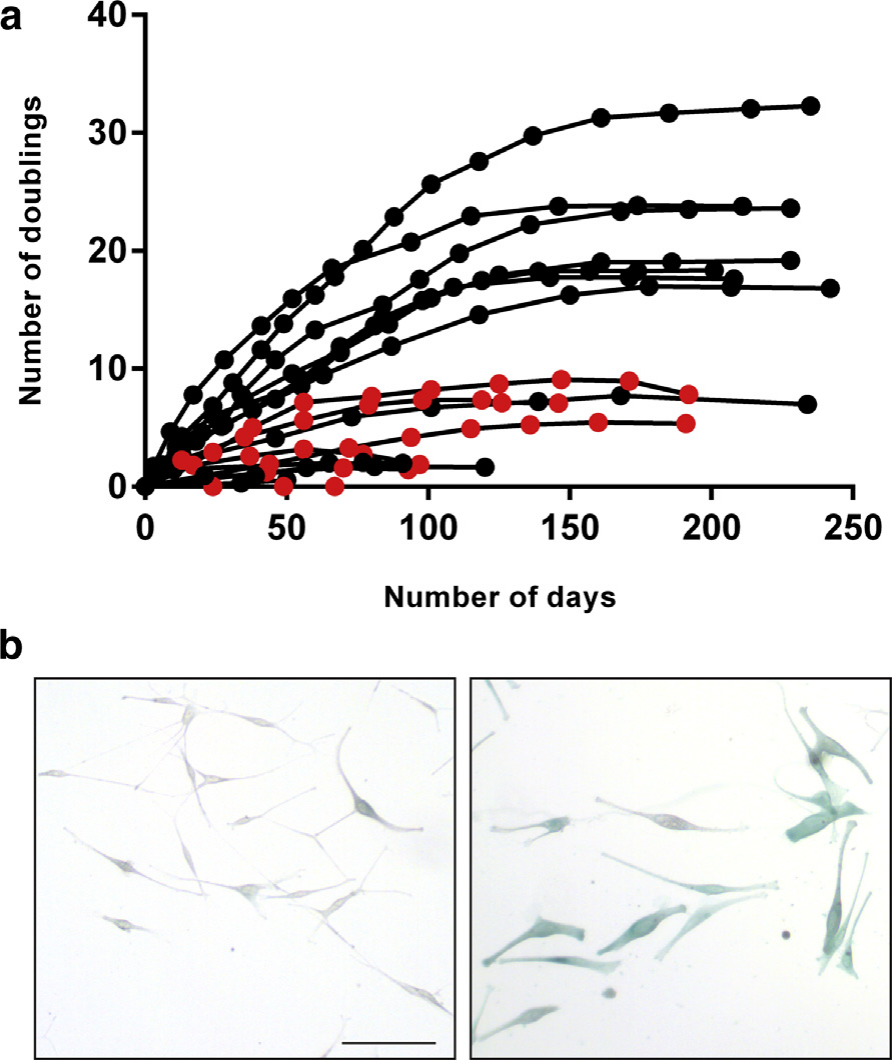
**Delayed cellular senescence in melanocytes from MPM patients.** (**a**) Cumulative growth curves of melanocytes from MPM (black points, n = 10) and control SPM patients (red points, n = 8). The first subculture when cells were first counted was designated day 0. At each subsequent subculture, the fold increase in number of cells was converted to cumulative population doublings. Each point represents one subculture. See Supplementary Materials and Methods online. (**b**) Confirmation of cellular senescence with β-galactosidase immunocytochemistry. Bright-field optics. Left and right images show representative growing and senescent (high-passage) cells, respectively, from one MPM patient. Senescent cells display positive β-galactosidase activity (blue) and melanin (grey to black). Scale bar for both images = 100 μm. MPM, multiple primary melanoma; SPM, single primary melanoma.

**Figure 2:**
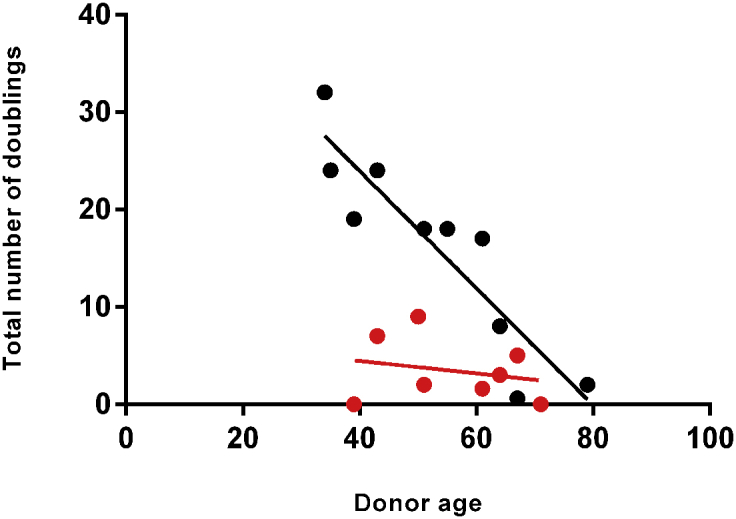
**Relationship of culture lifespan to donor age.** Scatterplot illustrating the relationship of culture lifespan with donor age from MPM (black points) and control SPM patients (red points). Color of regression line matches color of data points.
