# Supplementary material for: Common Delayed Senescence of Melanocytes from Multiple Primary Melanoma Patients
Source: J Invest Dermatol. 2017 Mar;137(3):766–8. doi: 10.1016/j.jid.2016.10.026 (PMC5319415; doi:10.1016/j.jid.2016.10.026)
Supplement: Supplementary Data [file mmc1.pdf]

SUPPLEMENTARY INFORMATION

METHODS

Primary culture and propagation of human melanocytes

Normal skin biopsies were obtained from patients with multiple or with a single melanoma upon written, informed consent, in the Surgery Day Hospital at the Fondazione IRCCS Istituto Nazionale Tumori (IFOM). A 5-mm punch biopsy of skin was taken under local anaesthetic and under aseptic conditions from the medial lower part of the arm. The biopsies were immediately placed in 15 ml sterile tubes full of chilled culture medium. Under sterile conditions, the biopsies were then transferred into another 15 ml tube with culture medium at pH 7 and sent on ice by next day delivery to St George's, University of London, where they were processed for cell culture.

Under sterile conditions, subcutaneous fat was removed from skin biopsies using a scalpel. Biopsies were briefly washed in penicillin/streptomycin (Sigma) and PBSA (Dulbecco's PBS without $MgCl_2$ and $CaCl_2$). Biopsies were then placed in two 5 ml volumes of 50 µg/ml gentamicin (Sigma) for 10 minutes each. Biopsies were then placed, epidermal side down, in 5 ml of 10 mg/ml dispase (Sigma) in Hank's balanced salt solution (Sigma) and kept overnight at $4^{O}C$.

The epidermis was peeled from the dermis the next day using sterile forceps. To ensure no possible contamination of dermal fibroblasts, forceps used to peel the epidermis never came into contact with the dermis. The epidermal sheet was cut into smaller fragments with a sterile blade and placed in 2.5 ml of 500 µg/ml trypsin (Sigma) in PBSA at $37^{O}C$ for 10 minutes. An epidermal suspension was made by continual shearing of the epidermis with a 21 gauge needle, attached to a 1 ml syringe. The suspension was centrifuged at 1,500 rpm for 15 minutes and the pellet was resuspended in 5 ml of growth medium. Primary cells were plated on to $2.5x10^5$ mitomycin C inactivated XB2 keratinocyte feeder cells in a T25 flask. Generation of mitomycin C inactivated XB2 feeder cells were carried out exactly as previously described (Soo et al., 2011). Components of melanocyte medium, as well as

protocols for passaging and generation of growth curves were exactly as previously described (Soo et al., 2011).

Institutional approval was obtained at both sites. The study was reviewed and approved by the Institutional Review Board and the Independent Ethics Committee at Istituto Nazionale Tumori (ref. INT124/13), and by the West London & GTAC Research Ethics Committee (ref. 06/Q0803/39) for the cell culture and storage procedures.

β-galactosidase immunocytochemistry

Detection of β-galactosidase activity was carried out exactly as previously described (Soo et al., 2011).

Statistical analysis

Significance of the difference between total lifespans of melanocytes from MPM and SPM patients was tested using a one-tailed Mann-Whitney U test. Presence of a significant relationship between culture lifespan and donor age was tested using an F test for linear regression. An analysis of covariance was used to test whether slopes of regression lines were significantly different. Significance of the difference between the mean ages of MPM and SPM patients was calculated with a two-tailed t-test. All significance tests were calculated using GraphPad Prism version 6 software.

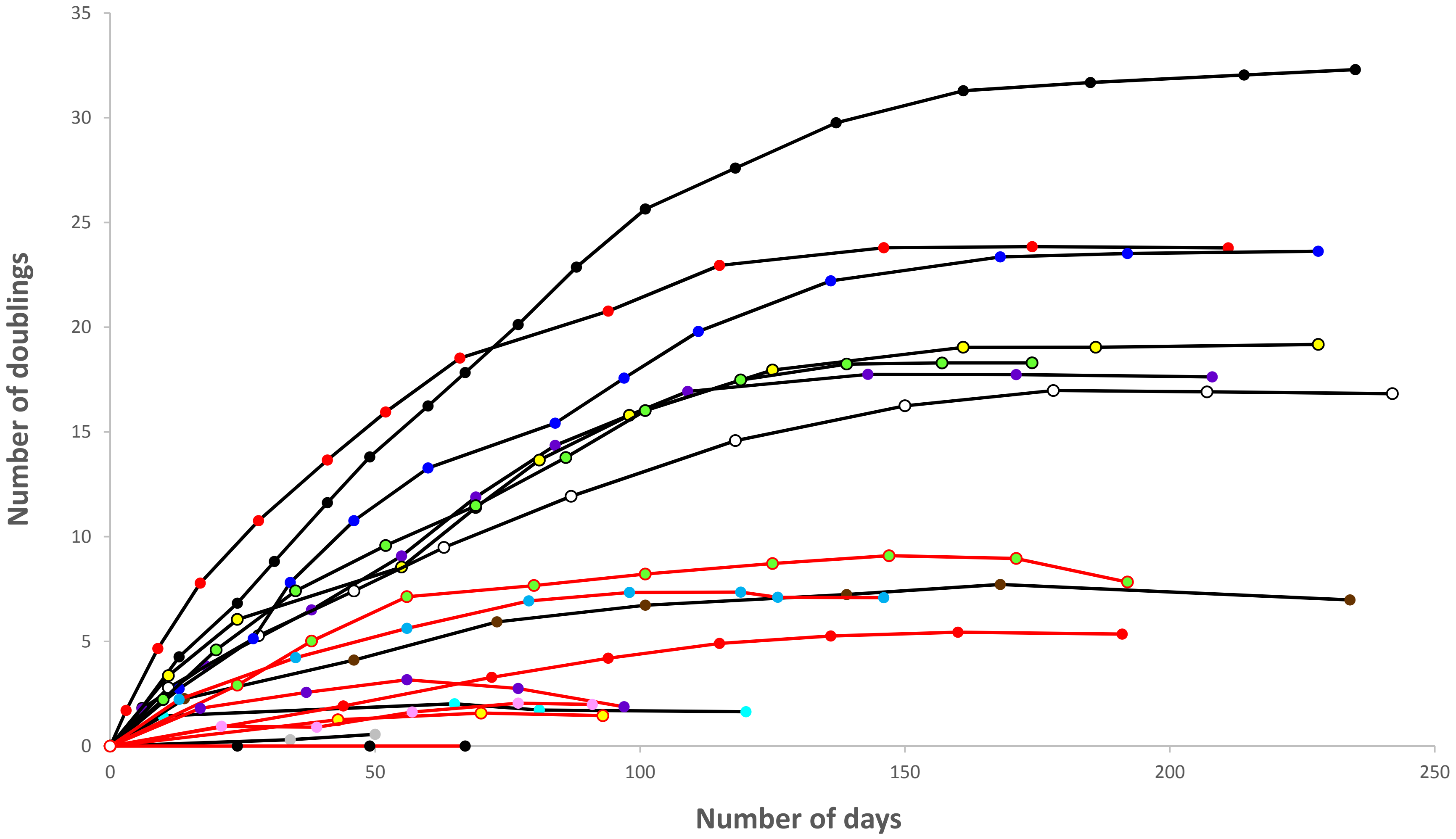

Figure S1. Cumulative growth curves of melanocytes from MPM and SPM patients. The same data as in Figure 1a, but in higher resolution, and here symbols are color coded and individual patient origin for each curve is identified in Table S2.

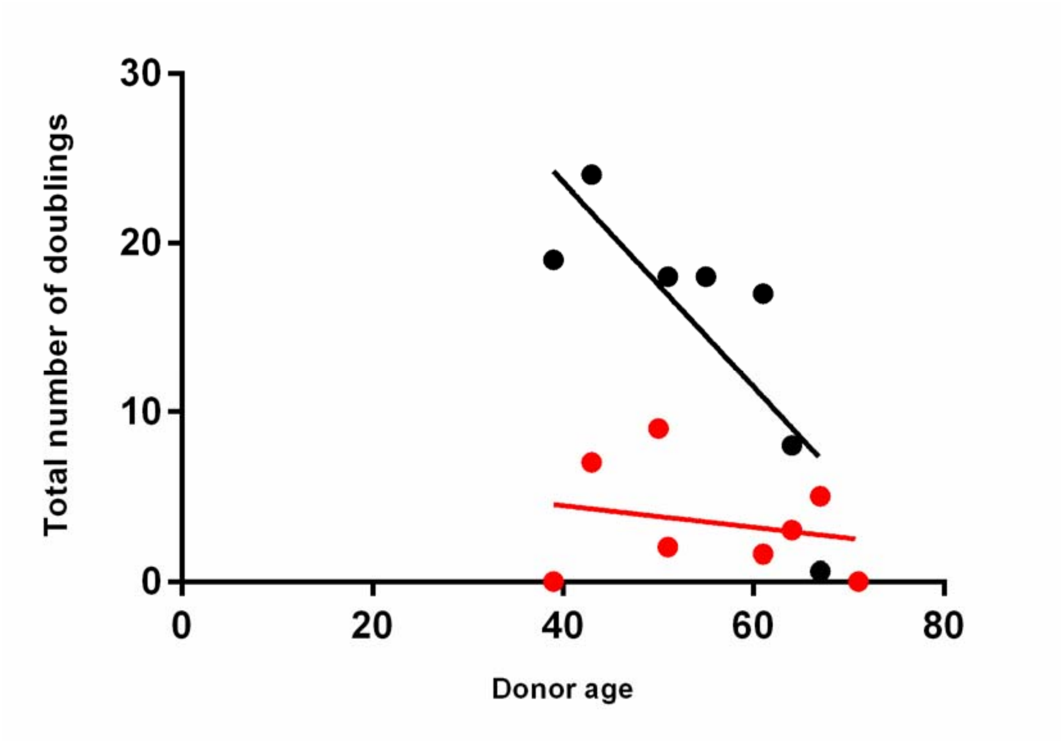

Figure S2. Culture lifespans from MPM patients within the same age range as SPM patients.

Scatter plot illustrating the relationship of culture lifespan to donor age from MPM (black points) and control SPM patients (red points). Colors of regression lines match color of data points.

Multiple Melanoma

Patient ID	Lifespan (doublings)[1]	Gender	Age	*CDKN2A* Promoter/5' UTR[2]	*CDKN2A* Exon 1α-2	*CDKN2A* Exon 3	*CDKN2A* Exon 1ß	*CDK4* Exon 2	*MITF* *E318K*	*MC1R*	*POT1* Exon 10	*TERT* Promoter
3M5	2	Male	79	-191 A/G	WT	540 C/T	WT	WT	WT	G248V; D294H	WT	WT
3M6	32	Male	34	-493 A/T	WT	WT	WT	WT	WT	WT	WT	-245 T/C
3M14	0.6	Male	67	WT	WT	WT	WT	WT	WT	WT	WT	-245 T/C
3M15	18	Male	55	WT	WT	540 C/T	WT	WT	WT	R160W;(I264I)	WT	-245 T/C
3M16	24	Female	35	WT	WT	WT	WT	WT	WT	R151C	WT	-245 T/C
3M20	19	Female	39	-191 G/G[3]	WT	WT	WT	WT	WT	V60L	WT	-269 G/A
3M22	17	Female	61	-191 A/G; -735G/A	WT	500 C/G	WT	WT	WT	V60L	WT	WT
3M23	8	Male	64	WT	WT	WT	WT	WT	WT	D294H	WT	WT
3M24	24	Male	43	-191 A/G	WT	WT	WT	WT	WT	WT	WT	WT
3M50	18	Male	51	-191 A/G; -735G/A	WT	500 C/G	WT	WT	WT	R151C	WT	WT

Single Melanoma

Patient ID	Lifespan (doublings)[1]	Gender	Age	*CDKN2A* Promoter/5' UTR[2]	*CDKN2A* Exon 1α-2	*CDKN2A* Exon 3	*CDKN2A* Exon 1ß	*CDK4* Exon 2	*MITF* *E318K*	*MC1R*	*POT1* Exon 10	*TERT* Promoter
3M36	9	Female	50	-191 A/G; -735G/A	WT	500 C/G	WT	WT	WT	V92M; R142H	WT	WT
3M37	1.6	Female	61	-735G/A	WT	500 C/G	WT	WT	WT	V92M; R160W	WT	WT
3M38	5	Female	67	WT	WT	WT	WT	WT	WT	V92M; (T314T)	WT	WT
3M51	0	Male	71	- 191 A/G	WT	WT	WT	WT	WT	WT	WT	WT
3M52	7	Male	43	- 191 A/G	WT	WT	WT	WT	WT	V60L	WT	WT
3M58	3	Male	64	WT	WT	WT	WT	WT	WT	R142H; R151C	WT	-245 C/C[3]
3M59	2	Male	51	WT	WT	WT	WT	WT	WT	V60L	WT	WT
3M60	0	Male	39	- 191 A/G	WT	WT	WT	WT	WT	R160W	WT	WT

Table S1. Genotypes of known familial melanoma genes, with gender, age and melanocyte culture lifespan, of MPM and SPM patients in our study.

Genotyping was carried out by sequence analysis according to published protocols (Bruno et al, 2016; Shi et al, 2014; Horn et al 2013).

1 Lifespan of normal epidermal melanocyte culture from each patient, in population doublings.

2 Polymorphisms found at positions -493 and -735 are located in the promoter while that at position -191 is located in the 5' UTR.

3 Polymorphisms are homozygous in these patients.

Multiple Melanoma

Patient ID	Growth curve symbol
3M5	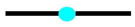
3M6	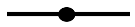
3M14	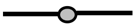
3M15	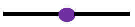
3M16	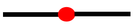
3M20	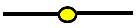
3M22	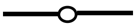
3M23	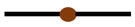
3M24	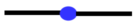
3M50	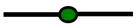

Single Melanoma

Patient ID	Growth curve symbol
3M36	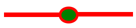
3M37	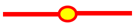
3M38	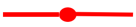
3M51	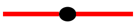
3M52	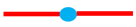
3M58	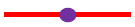
3M59	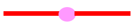
3M60	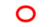

Table S2. Color key to donor patient for each melanocyte growth curve in figure S1.

REFERENCES

Soo JK, Mackenzie Ross AD, Kallenberg DM, Milagre C, Heung Chong W, Chow J, et al. Malignancy without immortality? Cellular immortalization as a possible late event in melanoma progression. Pigment Cell Melanoma Res 2011;24:490-503.

Bruno W, Pastorino L, Ghiorzo P, Andreotti V, Martinuzzi C, Menin C, et al. Multiple primary melanomas (MPMs) and criteria for genetic assessment: MultiMEL, a multicenter study of the Italian Melanoma Intergroup. J Am Acad Dermatol 2016;74:325-32.

Shi J, Yang XR, Ballew B, Rotunno M, Calista D, Fargnoli MC, et al. Rare missense variants in POT1 predispose to familial cutaneous malignant melanoma. Nat Genet 2014;46:482-6.

Horn S, Figl A, Rachakonda PS, Fischer C, Sucker A, Gast A, et al. TERT promoter mutations in familial and sporadic melanoma. Science 2013;339:959-61.
